# HRC promotes anoikis resistance and metastasis by suppressing endoplasmic reticulum stress in hepatocellular carcinoma

**DOI:** 10.7150/ijms.60610

**Published:** 2021-06-26

**Authors:** Suhong Xia, Jingwen Wu, Wangdong Zhou, Mingyu Zhang, Kai Zhao, Dean Tian, Jingmei Liu, Jiazhi Liao

**Affiliations:** 1 Department of Gastroenterology, Tongji Hospital of Tongji Medical College, Huazhong University of Science and Technology, Wuhan 430030, Hubei Province, China.; 2 Institute of Liver and Gastrointestinal Diseases, Tongji Hospital of Tongji Medical College, Huazhong University of Science and Technology, Wuhan 430030, Hubei Province, China.

**Keywords:** hepatocellular carcinoma, metastasis, HRC, endoplasmic reticulum stress, anoikis resistance

## Abstract

Histidine-rich calcium binding protein (HRC) is markedly overexpressed in hepatocellular carcinoma (HCC) and is significantly correlated with metastasis. Anoikis resistance and endoplasmic reticulum (ER) stress may have a critical effect on survival before metastasis. However, the potential functions of HRC in anoikis resistance in HCC remain unknown. Here, we uncovered the clinical value of HRC and its functional significance on anoikis in HCC. The positive expression of HRC was observably correlated with tumor size, tumor encapsulation, and tumor-node-metastasis (TNM) stage. The expression of HRC increased in HCC cells cultured in suspension. HRC enhanced the anoikis resistance of HCC, and promoted the HCC metastasis *in vivo*. Mechanistically, the anoikis resistance was probably dependent on endoplasmic reticulum stress. Modulating HRC level changed the ERS to affect anoikis resistance by acting protein kinase RNA-like ER kinase (PERK)-eIF2a-ATF4-CHOP signaling axis. In conclusion, we define HRC as a novel candidate oncogene involved in anoikis resistance and HCC metastasis, and provide a new potential therapeutic target for HCC.

## Introduction

Hepatocellular carcinoma (HCC) has become the third leading cause of cancer death all over the world 1. Although a large number of genes are associated with HCC metastasis, the underlying mechanism of HCC metastasis is still unclear. Therefore, understanding the mechanisms of HCC metastasis and investigating novel effective strategies are urgently required.

Attachment to the extracellular matrix (ECM) is crucial for cell anchorage, cell proliferation, differentiation, migration and survival functions 2. Anoikis involves anchorage-dependent programmed cell death caused by lack of cell detachment from ECM 3. Anoikis prevents the cell from disseminating to distant organs to limit cancer progression 4. Thus, malignant aggressive tumor cells develop different mechanisms to counter anoikis and obtain the ability to escape from the primary sites and migrate to distant organs or lymph nodes. Tumor cells decrease the down-regulation of pro-apoptotic proteins such as caspase-3 and increase the anti-apoptotic proteins such as Bcl-2, which will gain anoikis resistance 5, 6. Thus, anoikis resistance is a significant step for metastatic colonization and tumor progression, whereas the detailed mechanism of anoikis resistance in HCC is still unknown.

The histidine-rich calcium binding protein (HRC), as a sarcoplasmic reticulum protein, mainly regulates the Ca^2+^-release and Ca^2+^-absorption of the sarcoplasmic reticulum to maintain the Ca^2+^ - homeostasis of the sarcoplasmic reticulum 7. Previous studies have shown that HRC promotes tumor invasion and migration, and enhances the activation, proliferation and migration of hepatic stellate cells through endoplasmic reticulum (ER) stress 8, 9. Nevertheless, the exact role of HRC in anoikis resistance of HCC remains to be explained at present.

The endoplasmic reticulum (ER) is responsible for the synthesis, folding and maturation of certain proteins 10. ER stress usually occurs after unexpected disturbances such as physiology or pathology 11. Substantial evidence has clarified that ER stress is involved in multiple types of tumors and plays a pivotal role in mediating tumor survival and death 12-14. Moreover, we previous study had shown that ER stress- mediated cell growth and apoptosis inhibition 9. However, it is not clear whether it is related to anoikis resistance.

In the current study, we have uncovered that HRC promoted the progression and metastasis of human HCC cells *in vitro* and *in vivo*. Moreover, we revealed a significant role of HRC in anoikis resistance. The ER stress-involved PERK-ATF4-CHOP signaling pathway may release survival inhibitory signals after detachment. In a word, we hope that HRC becomes a potentially promising target for HCC.

## Materials and Methods

### Cell culture

All human HCC cell lines (Institute of liver diseases, Tongji Hospital of Tongji Medical College, Huazhong University of Science and Technology, Wuhan, Hubei, China) were cultured in Dulbecco's Modified Eagle Medium (DMEM) at 37°C in a 5% CO2 incubator. The medium contained with 10% FBS, 100μg/ml penicillin, and 100μg/ml streptomycin.

### HCC specimens

This study was approved by the Ethics Committee of Tongji Medical College. All HCC specimens were provided informed consent that was obtained in compliance with the guidelines of the Declaration of Helsinki. The HCC cohort contained 86 adult patients with HCC who underwent curative resection between 2009 and 2012 at the Tongji Hospital of Tongji Medical College (Wuhan, China).

### Construction of tissue microarrays and immunohistochemistry

HCC samples and the corresponding adjacent liver tissues were performed to construct a tissue microarray (Shanghai Biochip Co., Ltd. Shanghai, China). Immunohistochemistry was performed on 4-μm-thick, routinely processed paraffin-embedded sections. After baking at 60 °C for an hour, the tissue sections were deparaffinized with xylene and dehydrated through gradient ethanol immersion. Then the endogenous peroxidase activity was blocked with 3% (vol/vol) hydrogen peroxide. After following by three 3-min washes with phosphate-buffered saline (PBS), the slides were immersed in 0.01 mol/L citrate buffer solution (pH 6.0) and placed in a microwave oven for 30 min. After washing in PBS (pH 7.4, 0.01 mol/L), the tissue sections were incubated with the primary antibody HRC (ATLAS ANTIBODIES, HPA004833, 1:1000) in a moist chamber at 4 °C overnight. After three 5 min washes with PBS, the peroxidase-conjugated second antibody (Santa Cruz) was used to incubate the sections for 30 min at room temperature, followed by additional three 5 min washes with PBS. The reaction product was observed with diaminobenzidine for 2 min. Images were used by a light microscope (Olympus, Japan) equipped with a DP70 digital camera.

### Reagents and antibodies

The ER stress inducer thapsigargin (TG) and inhibitor 4-phenylbutyric acid (4-PBA) were purchased from Cayman Chemical (Ann Arbor, MI, USA). The antibodies are shown in Supplementary Table S1.

### RNA interference and construction of lentivirus for stable cell lines

For RNA interference, the sequence of small interfering RNA (siRNA) oligonucleotides (synthesized by RiboBio, Guangzhou, China) were used as follows: (siATF4 forward, 5′‐AGGAGCAAAACAAGACAGCATTTT‐3′ and reverse, 5′-ATGCTGTCTTGTTTTGCTCCTTTT‐3′; and siSERCA2 forward, 5′‐CTGTCCATGTCACTCCACTTCC‐3′ and reverse, 5′‐AGCGGTTACTCCAGTATTGCAG‐3′. A scrambled siRNA was used as negative control. All constructs were transfected into cells using lipofectamine 3000 (Invitrogen) according to the manufacturer's instructions. Lentiviruses encoding short hairpin (sh)RNAs were produced using pLKO.1-puro (Genechem, Shanghai, China and DesignGene, Shanghai, China) and were denoted as “shHRC.” Recombinant lentivirus overexpressing HRC and CHOP was constructed using pLKO.1-puro and pLKO.1-neo (DesignGene Biotechnology, Shanghai, China) and was denoted as “Lv-HRC” and “Lv-CHOP” Cells were transfected using Lipofectamine 3000 (Invitrogen, CA, USA) per the manufacturer's instructions. In brief, cells were transfected with lentivirus at a multiplicity of infection of 10-30 for 12 h. The medium was replaced with DMEM containing 10% FBS and the cells were cultivated for another 24 h. Transfected cells were selected with puromycin or G418 for 2 weeks. Target gene expression was confirmed by both western blotting and real-time reverse transcription RT-qPCR.

### RNA extraction and real-time RT-PCR

Total RNA was extracted using TRIzol reagent (Invitrogen, Carlsbad, CA, USA) according to the manufacturer's instructions. Reverse-transcribed complementary DNA was synthesized using the PrimeScript RT Reagent Kit (TaKaRa, Ostu, Japan). Real-time PCR was run using SYBR Premix ExTaq (TaKaRa, Ostu, Japan) on ABI StepOne Real-Time PCR System (Applied Biosystem, Carlsbad, CA, USA). The thermal cyclers were as follows: 40 cycles of 95°C for 30 s, 60°C for 5 s, and 70°C for 30 s. The value of 2^-ΔΔCt^ was used to determine fold differences between samples. The sequences of the primers used for RT-qPCR are listed in Supplementary Table S2.

### Western blotting analysis

Western blotting was performed as previously described 9. Tissues and cell lysates were harvested in ice-cold RIPA lysis buffer supplemented with protease and phosphatase inhibitors. Briefly, proteins were separated by SDS-PAGE and transferred to polyvinylidene difluoride membrane. Nonspecific binding sites were blocked with 5% non-fat milk for one hour. The membranes were incubated with primary antibodies overnight at 4 °C. Western blotting of GAPDH on the same membrane was used as a loading control. The membranes were then washed with TBST 3 times and incubated with an HRP-conjugated secondary antibody. Proteins were visualized using an ImmobilonTM Western Chemiluminescent HRP substrate (Millipore, USA). The primary antibodies used are listed in Supplementary Table S1.

### Anoikis assay

The Adherent culture was maintained on Petri dishes (Corning). To induce anoikis under anchorage-independent condition, culture plates were coated with poly-HEMA (10 mg/ml, Sigma). The assay was performed as previously described 15. This is a well-established method of studying anoikis and dependents on the nonionic nature of poly-HEMA to prevent matrix deposition and subsequent cell attachment. Approximately 1
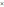
10^6^ cells were plated in poly-HEMA-coated dishes. After the expected treatment time 48h was reached, the suspended HCC cells were centrifuged and divided equally into dishes or wells for adherent culture. These viable cells which survived in three-round anoikis induction, were stored and termed as anoikis-resistant cells.

### Caspase-3 assays

Caspase-3 assays were performed as previously described 9, with slight modifications. Briefly, the HCC cells were cultured at 3
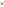
10^5^ cells ⁄ well in a 24-well plate under serum- free conditions in suspension.

### Apoptosis analysis

For cell apoptosis assays, apoptosis cells were measured by a FITC Annexin V Apoptosis Detection Kit I (BD Pharmingen, San Diego, CA, USA) according to the manufacturer's protocol. Cells were collected under anchorage-independent conditions, washed twice with PBS and resuspended in 200 µL binding buffer. Cells were stained with 5 µL Annexin V and 5 µL propidium iodide (PI), and incubated for 30 min at 4°C in the dark. Then, cells were analyzed by flow cytometer.

### *In-vivo* metastasis assay and bioluminescence imaging

All animal procedures were carried out in accordance with the Guide for the Care and Use of Laboratory Animals and standards articulated in the Animal Research: Reporting of *In Vivo* Experiments. All experimental animals were approved by the experimental animal ethics committee of Tongji Medical College of Huazhong University of Science and Technology. To metastatic HCC model, the BALB/c nude mice (5-week-old, male) were divided into four groups (n=10, respectively) at random. After that, 6×10^6^ cells were suspended in PBS and mixed with Matrigel (BD Biosciences, CA, USA), then injected orthotopically into the left liver lobes of nude mice. Nine weeks later, D-Luciferin (Gold Biotechnology, USA) was injected intraperitoneally into each mouse to monitor tumor formation and metastasis, and images were captured with Lago X optical imaging system (SI Imaging, USA). Lung tissues were excised after 9 weeks, and fixed with 4% paraformaldehyde, and stained with hematoxylin and eosin in order to count metastatic nodules at the next step.

### *In-vitro* migration and invasion assays

Cell migration and invasion assay were performed as previously described 16. Briefly, a 24 well chamber with 8-μm pore filter (Corning corporation, USA) was used. For migration assay, 5×10^4^ cells were seeded into the upper chamber in serum-free medium. For invasion assay, chamber inserts were coated with 200 mg/ml of Matrigel and dried overnight under sterile conditions. Then, 1×10^5^ cells were plated in the top chamber. The mean of triplicate assays for each experimental condition was used.

### Statistical analysis

All data in this study were recorded as the mean ± standard deviation (sd). P values were statistically performed by the χ2 test for categorical variables between the two groups and by Student's test for quantitative data. Parametric One-Way analysis of variance (ANOVA) test was used for comparisons between more than two groups. Statistical values were analyzed with SPSS22.0 software (SPSS Inc., Chicago, IL, USA) and a P-value ≤ 0.05 was considered as statistically significant.

## Results

### HRC is significantly upregulated in human HCC tissues

**Figure 1:**
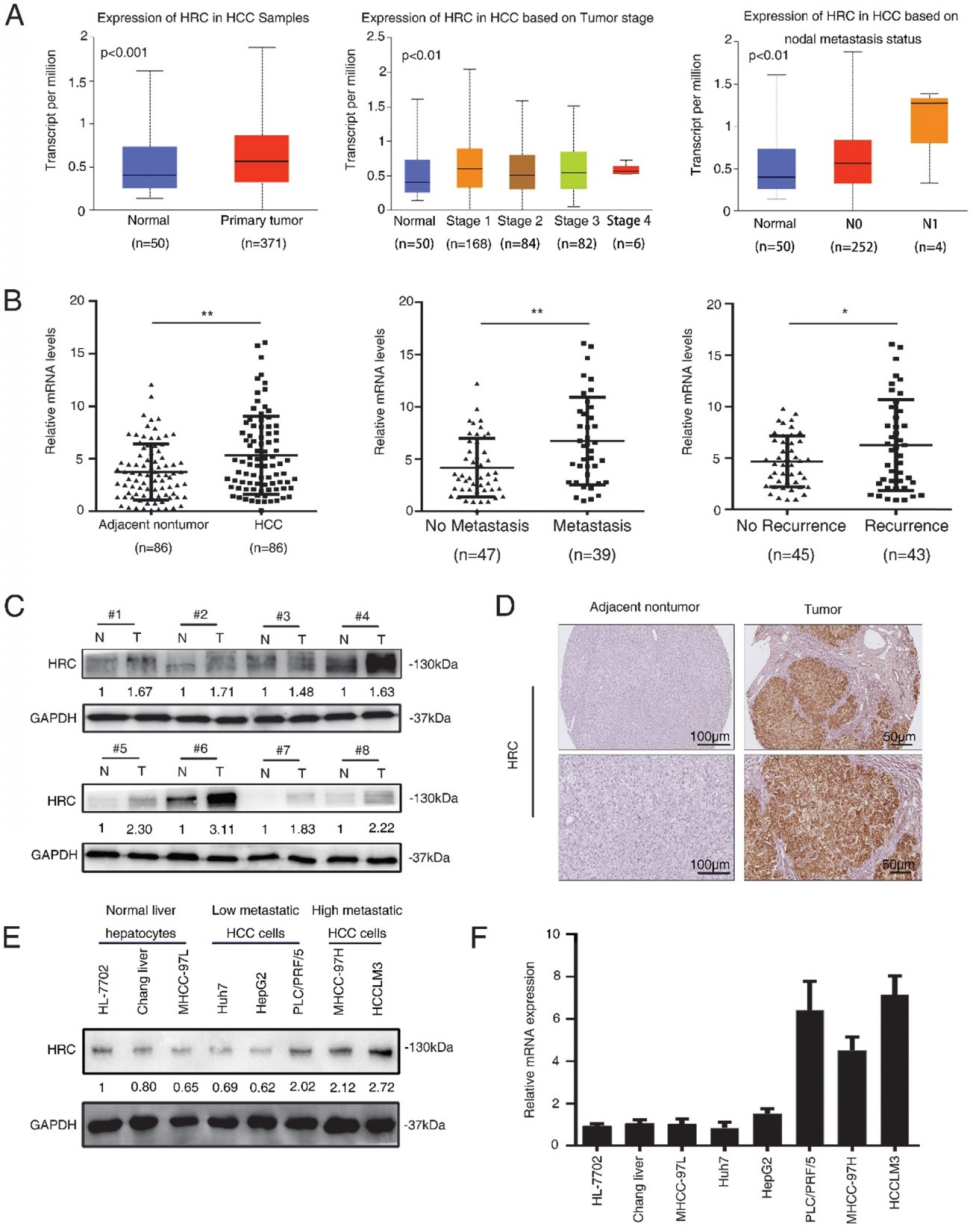
** HRC is significantly upregulated in human HCC tissues.** (A) Expression level of HRC in 371 HCC tissues compared with 50 normal liver tissue samples in the TCGA database. HRC expression was positively associated with the clinical stage of HCC. The expression of HRC was correlated with lymph node metastasis in HCC patients. **P<0.01, ***P<0.001. (B) Relative HRC mRNA expression in 86 paired HCC and adjacent nontumorous tissues. Relative HRC mRNA expression in HCC samples with metastasis (n=39) or without metastasis (n=47). Relative HRC mRNA expression in HCC samples with recurrence (n=43) or without recurrence (n=45). **P<0.01, *P<0.05. (C) Western blot analysis of HRC expression levels in 8 matched pairs of HCC and adjacent nontumorous tissues. (D) Representative image of HRC expression with IHC staining in adjacent nontumorous tissues and HCC tissues. (E) Western blot analysis of expression level of HRC in normal liver cell and different HCC cell lines. (F) Relative mRNA of HRC in normal liver cell and HCC cell lines. Data represent mean ±SD from three independent experiments, * P<0.05.

To investigate the correlation between HRC expression and clinical characteristics of HCC. We firstly downloaded the expression and clinical data from the TCGA database for further analysis. The expression level of HRC was upregulated in 371 HCC tissues compared with that in 50 normal liver tissue samples according to the TCGA database, and the data showed that HRC was significantly correlated with the degree of malignancy of HCC including tumor stage and lymph node metastasis (Figure 1A). Furthermore, HRC mRNA expression was analyzed in paired HCC tissues and adjacent nontumor tissues by RT-qPCR. HRC was higher in the HCC tissues than in the adjacent nontumor tissues, and patients with metastatic (n=39) or recurrent (n=43) HCC had likewise higher than those with nonmetastatic (n=47) or nonrecurrent (n=45) tissues (Figure 1B). In addition, HRC expression was significantly correlated with tumor size, tumor encapsulation and higher tumor-nodule-metastasis (TNM) stage (Table 1).

To confirm the overexpression of HRC in HCC, the protein level of HRC was examined in 8 paired human HCC samples and nontumor tissues by western blot. A similar result was observed at the protein level of HRC (Figure 1C). The same trend in HRC elevated in the HCC tissues was also confirmed by IHC (Figure 1D). We then detected HRC expression in HCC cell lines and found that HRC level was higher in HCC cells with high metastatic capability (Figure 1E, F). These data demonstrated that HRC frequently upregulated in HCC samples and metastatic HCC cells, and suggesting that it might be a decisive role in HCC progression.

### HRC elevates anoikis resistance and metastatic ability to hepatocellular carcinoma cells

**Figure 2:**
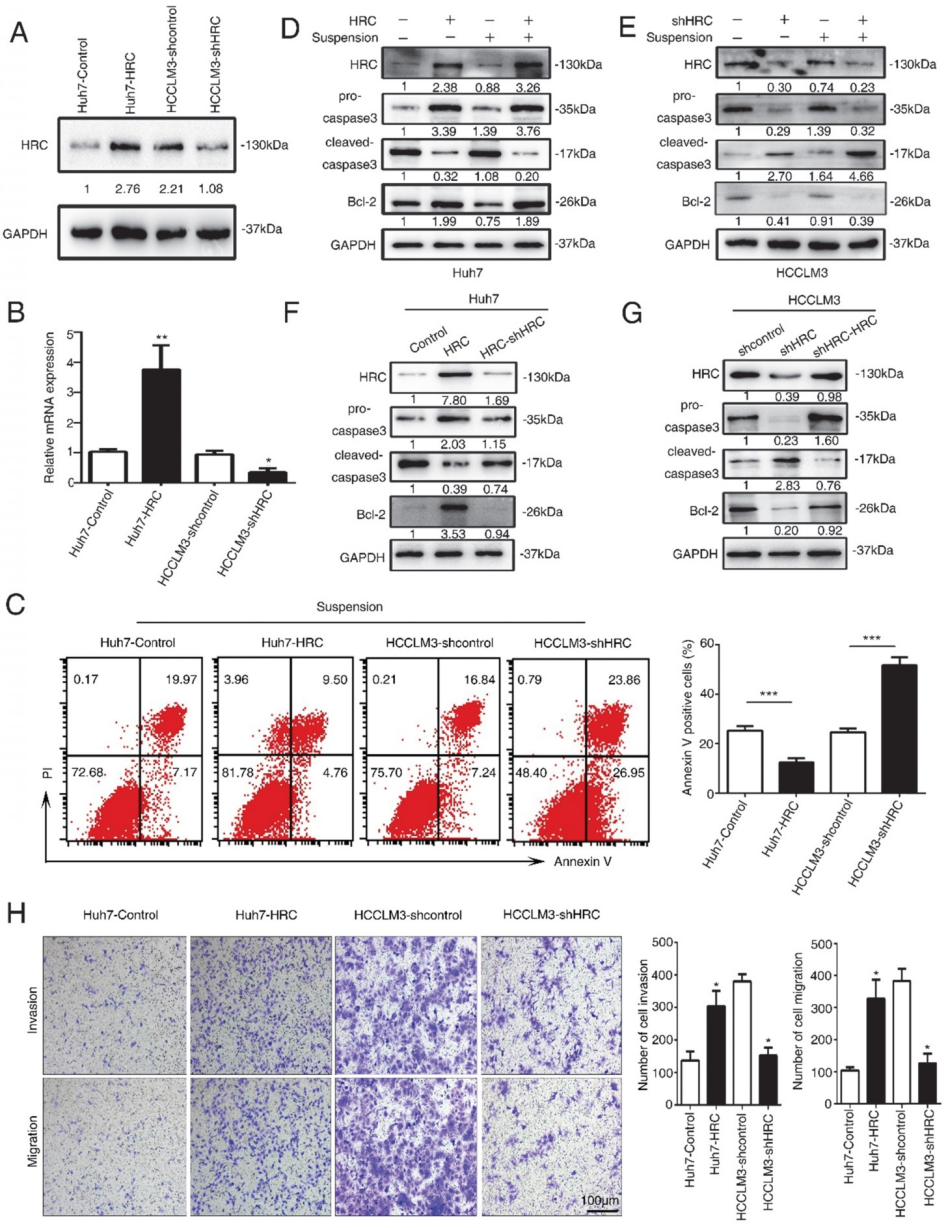
** HRC elevates anoikis resistance and metastatic ability to hepatocellular carcinoma cells.** (A) Western blot analysis was used to show HRC expression in Huh7 and HCCLM3 cells with lentivirus transfection. (B) Relative mRNA HRC in Huh7 and HCCLM3 cells with lentivirus transfection. (C) HCC cells stably expressing HRC were treated with poly-HEMA for 48 h. Flow cytometry analysis of apoptosis was determined using Annexin V-FITC/PI staining. Data represent mean ±SD from three independent experiments, * P<0.05. (D) Western blot analysis was used to detect the cleaved-caspase3 and Bcl-2 as indicative of cell apoptosis in Huh7and (E) HCCLM3 cells were treated with poly-HEMA for 48 h. (F) HRC re-knockdown (G) and HRC-reconstituted cells were treated with poly-HEMA for 48 hours, then cells were harvested and analyzed by western blot using indicated antibodies. (H) Transwell assays were performed on anoikis-resistant cells to show the invasive and migratory abilities of HCC cells after changes of HRC expression for 24 h. The scale bar represents 100µm. Images shown are representative of at least three independent experiments. The data are shown as the mean ± SD; *P < 0.05.

**Figure 3:**
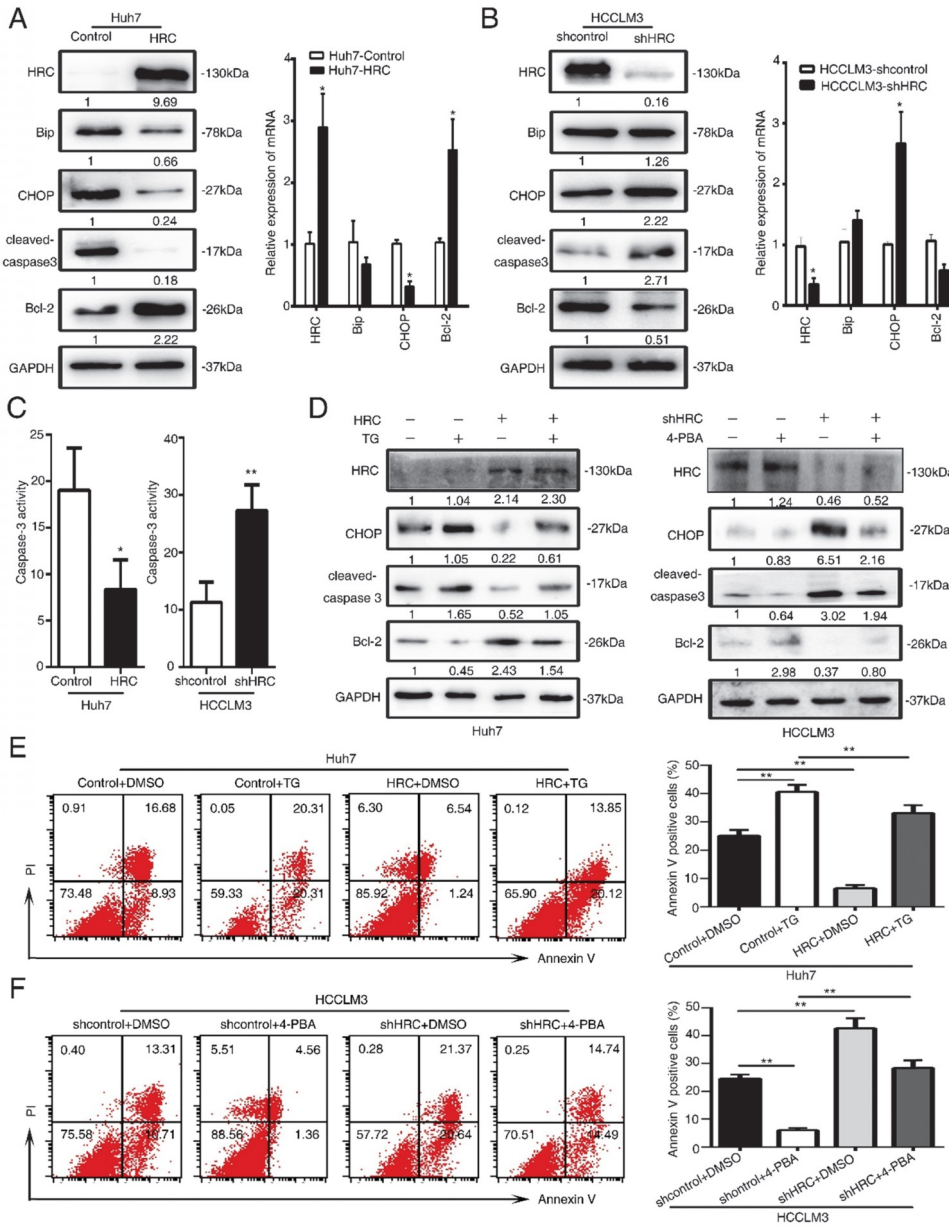
** HRC promotes anoikis resistance via suppressing endoplasmic reticulum stress.** (A, B) The expression of Bip, CHOP, cleaved-caspase3 and Bcl-2 were assessed by RT-qPCR and western blot after cells treated with poly-HEMA for 48 hours. Data represent mean ±SD from three independent experiments, * P<0.05. (C) Caspase3 activity of HCC cells were measured. Data represent mean ±SD from three independent experiments, * P<0.05, ** P<0.01. (D) Huh7 cells were pretreated with 1mM thapsigargin (TG) and HCCLM3 cells were pretreated with 1mM 4-phenylbutyrate acid (4-PBA) under detached condition for 48 h, the protein expression of CHOP, cleaved-caspase3 and Bcl-2 were assessed. (E, F) Huh7 cells were pretreated with 1mM thapsigargin (TG) and HCCLM3 cells were pretreated with 1mM 4-phenylbutyrate acid (4-PBA) under detached condition for 48 h. Cell apoptosis was measured by flow cytometry. Images shown are representative of at least three independent experiments. The data are shown as the mean ± SD; **P < 0.01.

To figure out the role of HRC in HCC development, Huh7 and HCCLM3 cells were selected for establish stable cell lines, Huh7-HRC and HCCLM3-shHRC, with lentivirus infection. Western blot and RT-qPCR analysis confirmed HRC overexpression and knockdown (Figure 2A, 2B). Anoikis resistance is an early step in cancer metastasis 17. HRC may play the important role in anoikis resistance and improve the ability of HCC to promote tumor metastasis. To verify this hypothesis, we showed the apoptotic trend of cells in suspension. Upregulated HRC cells and deficient cells were treated with poly-HEMA for 48h, and then were tested with flow cytometry using FITC-Annexin V apoptosis detection kit. As demonstrated in Figure 3A, the rate of anoikis cells was markedly increased in HRC-deficient cells and decreased in HRC-overexpression cells. Therefore, our results showed that upregulation of HRC contributes to anoikis resistance in HCC cells.

Anoikis is typically characterized by stimulating the activation of caspase-3 cleavage 18. To test the functions of HRC in anoikis resistance, we analyzed that caspase-3, cleaved capsese-3 and Bcl-2 expression in suspension. The upregulation of HRC effectively decreased the level of cleaved caspase-3 and increased the expression of Bcl-2 protein in Huh7-HRC cells during suspension (Figure 2D). The oppositive results were observed in HRC-deficient HCCLM3 cells during suspension (Figure 2E). The levels of cleaved caspase-3 and Bcl-2 were reversed in the cells silenced in Huh7-HRC cells during suspension (Figure 2F). Meanwhile, reconstitution of HRC into deficient cells overturn the previous results in suspension (Figure 2G). In the following section, transwell assay analysis indicated that overexpression HRC increased the migrative and invasive ability of Huh7 cells, while knockdown HRC in HCCLM3 cells decreased the invasion and migration (Figure 2H). In conclusion, these results showed that HRC had a decisive role in preventing HCC cells from anoikis.

### HRC promotes anoikis resistance via suppressing endoplasmic reticulum stress

The dual role of endoplasmic reticulum (ER) stress in survival and apoptotic signals remains controversial, and it is still unclear whether the protective elements or inhibitory factor 19, 20. In response to ER stress, the Unfolded Protein Response (UPR) cascade signal transduction reaction occurs to protect from damage 21. To understand its role in anoikis resistance, we analyzed that the critical protein expression in ER stress and apoptotic signals. The results showed that HRC overexpression significantly decreased the C/EBP homologous protein transcription factor (CHOP) and cleaved caspase-3 but increased the expression of Bcl-2 protein during suspension (Figure 3A). Moreover, HRC silence increased CHOP expression but decreased Bcl-2 protein in suspension (Figure 3B).

We further detected the activation of pivotal apoptosis of medium, caspase-3. Up-regulated HRC hindered whereas HRC knockdown strengthened caspase-3 activation (Figure 3C). To further evaluate the role of ER stress in anoikis resistance, we used the ER stress inducer, thapsigargin (TG) and an ER stress inhibitor, 4-phenylbutyrate acid (4-PBA). Interestingly, we found that the usage of ER stress inducer TG could not only enhance the expression of ER stress molecular indicators but pro-apoptotic proteins. Simultaneously, ER stress inhibitor 4-PBA showed the opposite discovery (Figure 3D). Next, flow cytometry was performed after pretreatment with TG or 4-PBA. The findings demonstrated that TG increased the rate of apoptosis and weakened the anti-apoptosis rate of HRC (Figure 3E). In contrast, treatment of 4-PBA had the opposite results (Figure 3F). These data indicated that ER stress was involved in HRC-mediated anoikis resistance inhibition.

### HRC suppresses endoplasmic reticulum stress by protein kinase RNA-like ER stress (PERK)-ATF4-CHOP signaling axis

**Figure 4:**
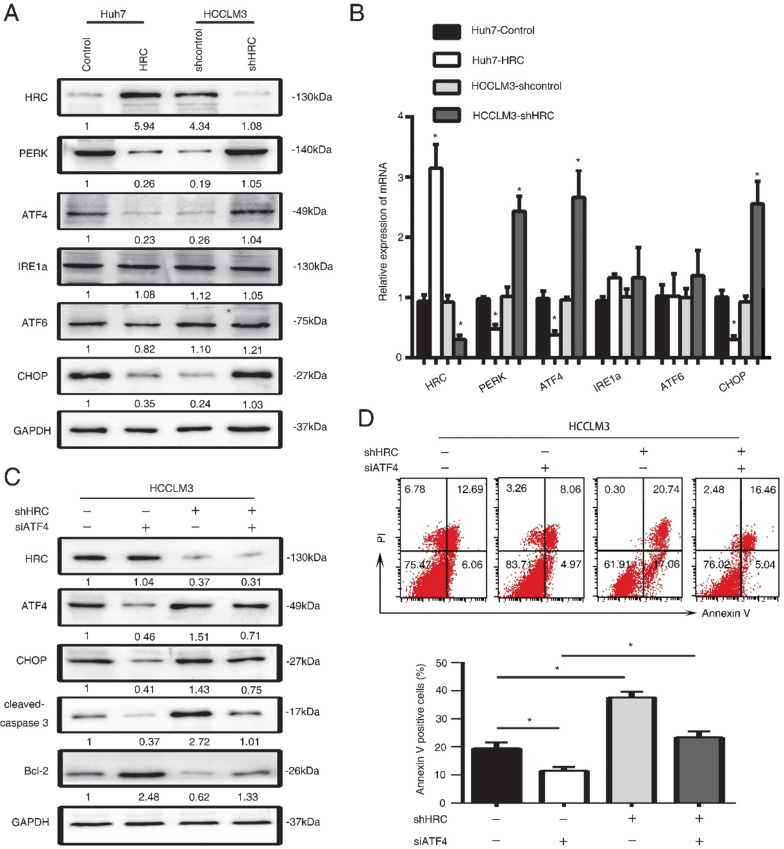
** HRC suppresses endoplasmic reticulum stress by protein kinase RNA-like ER stress (PERK)-ATF4-CHOP signaling axis.** (A) Western blot analysis was used to detect the expression of endoplasmic reticulum stress-associated proteins, including PERK, ATF4, ATF6, IRE1a, CHOP in HCC cells with poly-HEMA for 48 hours. (B) Relative mRNA the expression of endoplasmic reticulum stress-associated proteins, including PERK, ATF4, ATF6, IRE1a, CHOP in HCC cells with poly-HEMA for 48 hours. The data are shown as the mean ± SD; *P < 0.05. (C) HCCLM3 anoikis-resistant cells were transfected with ATF4 siRNA, western blot to detect the level of ATF4, CHOP, cleaved-caspase3 and Bcl-2. (D) Flow cytometry analysis of apoptosis was determined using Annexin V-FITC/PI staining in HCCLM3 anoikis-resistant cells expressing siATF4. Images shown are representative of at least three independent experiments. The data are shown as the mean ± SD; *P < 0.05.

**Figure 5:**
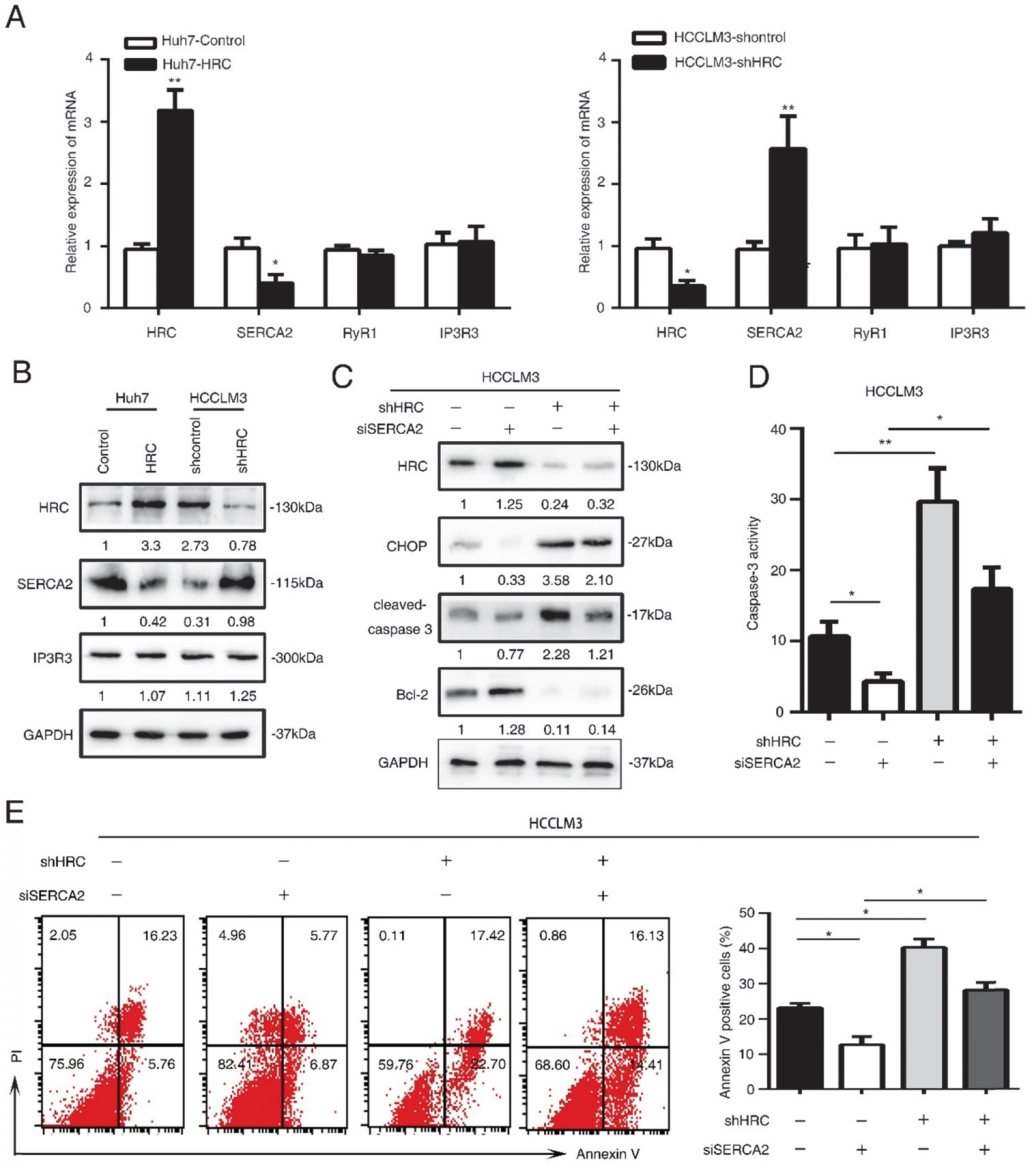
** SERCA2 is associated with the anoikis role of endoplasmic reticulum stress.** (A) Relative mRNA the expression of SERCA2, RYR1, IP3R3 in HCC cells with poly-HEMA for 48 hours. The data are shown as the mean ± SD; *P < 0.05. (B) Western blot analysis was used to detect the SERCA2 and IP3R3 in HCC cells with poly-HEMA for 48 hours. (C) HCCLM3 cells were transfected with SERCA2 siRNA, western blot to detect the level of CHOP, cleaved-caspase3 and Bcl-2. (D) Caspase3 activity were measured in HCCLM3 anoikis-resistant cells expressing SERCA2 siRNA. Data represent mean ±SD from three independent experiments, * P<0.05, ** P<0.01. (E) HCCLM3 anoikis-resistant cells were transfected with SERCA2 siRNA, cell apoptosis was analyzed by flow cytometry. Images shown are representative of at least three independent experiments. The data are shown as the mean ± SD; *P < 0.05.

ER stress has a critical role in cell apoptosis. There are three transducers at the ER membrane to sense the changes in stress, including the activating transcription factor 6 (ATF6), the PKR-like ER kinase (PERK) and the inositol-requiring enzyme 1 (IRE1) 22. Several pro-apoptotic genes and suppression of anti-apoptotic Bcl-2 proteins by the activating transcription factor 4 (ATF4)-CHOP signaling 23, 24. Therefore, we examined the three transducers in HCC cells, and found that the PERK, ATF4 and CHOP protein levels significantly decreased in HRC-upregulated cells (Figure 4A, 4B). In addition, to explore whether HRC suppressed ER stress and anoikis by PERK-ATF4-CHOP signaling, RNA interference (RNAi) was used to inhibit ATF4 in HCCLM3 cells. Interference with the expression of ATF4 could suppress the expression of CHOP and cleaved-caspase 3 but increased the level of Bcl-2, and reversed the effect on HRC downregulation on CHOP, cleaved-caspase 3 and Bcl-2 proteins (Figure 5C). Accordingly, ATF4 interference increased the survival of suspension cells and reversed the effect of the HRC knockdown on the survival of suspension cells (Figure 4D). From the above results, we concluded that HRC suppressed ER stress via PERK-ATF4-CHOP signal pathway.

### SERCA2 is associated with the anoikis role of endoplasmic reticulum stress

HRC is good candidate for regulating Ca^2+^ uptake, storage and release, so it may have an effect on Ca^2+^ homeostasis 25. Imbalance of Ca^2+^ homeostasis in the ER stimulates activation of ER stress responses 26. In our previous study, we found that HRC could increase [Ca2+]_i_ into ER by upregulating SERCA2. To explore whether HRC could regulate Ca^2+^ channels in the ER in suspension, we examined the expression of ryanodine receptor (RYR), inositol-1,4,5-trisphosphate receptor (IP3R), Sarco/endoplasmic reticulum calcium ATPase (SERCA). SERCA2 has significantly decreased in HRC overexpression Huh7 cells, but no noticeable changes were found in RyR1 and IP3R3 (Figure 4A, 4B). We noticed that SERCA2 was greatly upregulated in HRC knockdown HCCLM3 cells, but not in RyR1 and IP3R3 (Figure 5A, 5B). To further examine the functions of SERCA2 in ER-mediated anoikis, RNA interference (RNAi) was used to inhibit SERCA2 in HCCLM3 cells. As expected, interference with the expression of SERCA2 could suppress the expression of CHOP and cleaved-caspase 3, and reversed the effect on HRC downregulation on CHOP and cleaved-caspase 3 (Figure 5C). We further found that down-regulating the expression of SERCA2 significantly reduced the activity of caspase-3 (Figure 5D). Next, we investigated whether the interference of SERCA2 affects the rate of anoikis. Interestingly, the data suggested that the rate of anoikis was greatly decreased while interfering with SERCA2. In addition, the interference of SERCA2 could significantly reverse the effect of HRC silencing on the rate of anoikis (Figure 5E). Taken together, these data suggested that SERCA2 was involved in the process of ER stress-induced anoikis, and reversed the effect of HRC on ER stress.

### HRC promotes HCC metastasis by suppressed ER stress

**Figure 6:**
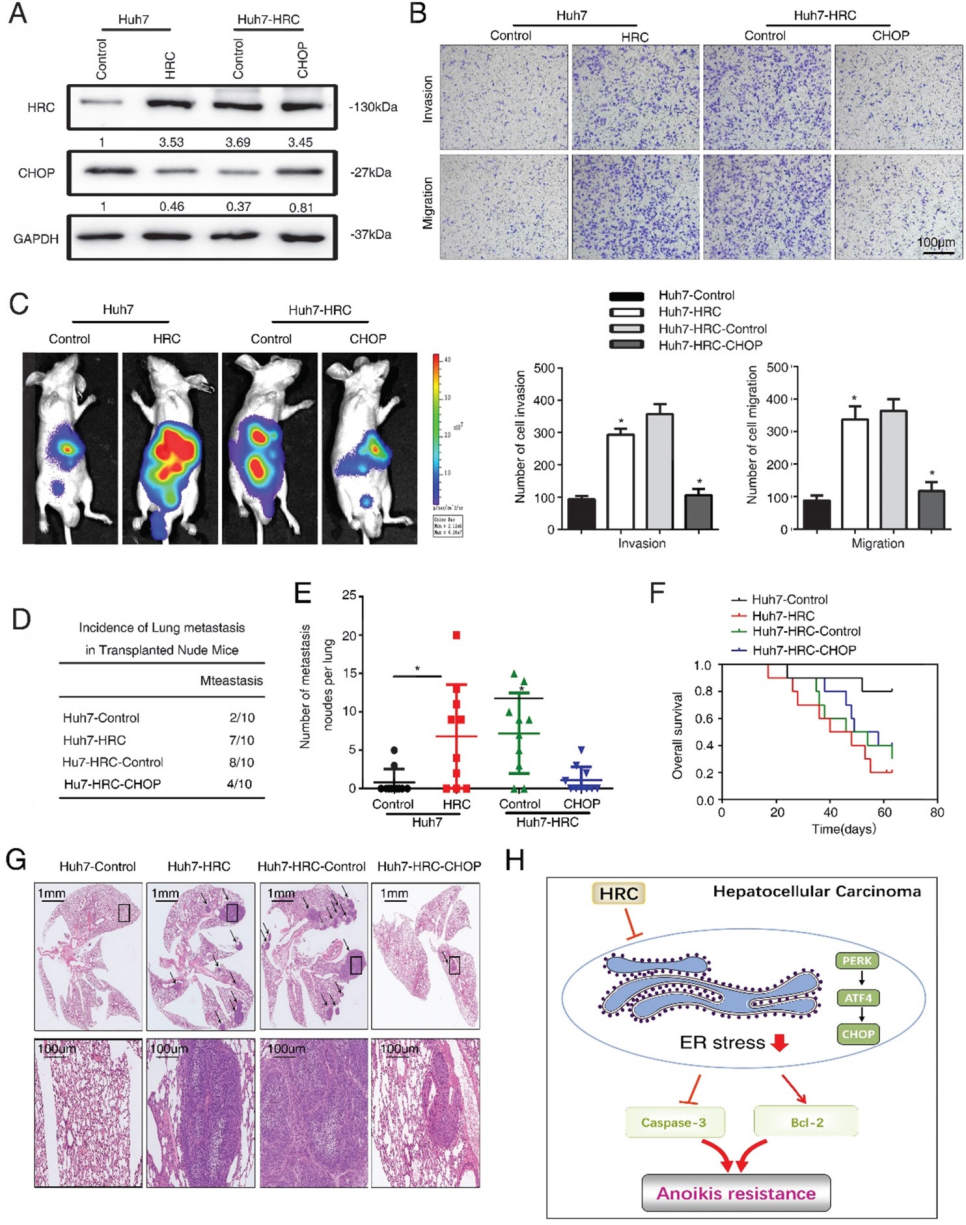
** HRC promotes HCC metastasis by suppressed ER stress.** (A) HCCLM3 anoikis-resistant cells were transfected with CHOP, western blot to detect the level of HRC and CHOP. (B) Transwell assays were performed on anoikis-resistant cells to show the invasive and migratory abilities of HCC cells after changes of CHOP expression for 24 h. The scale bar represents 100µm. Images shown are representative of at least three independent experiments. *P < 0.05. (C-G) *In vivo* assays shown that HRC-mediated ER stress can inhibit HCC metastasis. (C) Bioluminescent images in nude mice were injected with the indicated cells in the liver. (D) Incidence of lung colonization in the treated nude mice. (E) Number of metastatic nodules in lung. (F) Overall survival time of nude mice in different groups. (G) Representative HE staining images of lung tissues from the different groups were shown. The scale bars represent 1mm (upper panel) and 100 µm (lower panel). The data are shown as the mean ± SD. P < 0.05 was used as the significance threshold for comparisons between the different groups. (H) HRC promotes anoikis resistance and metastasis through suppressing endoplasmic reticulum stress in hepatocellular carcinoma.

Finally, we explored the role of ER stress in HCC metastasis. We ectopically overexpressed CHOP expression in Huh7-HRC cells (Figure 6A). Transwell assays showed that upregulation of HRC increased the migration and invasion abilities in Huh7 cells, whereas ectopic overexpression of CHOP inhibited the migration and invasion abilities induced by HRC overexpression (Figure 6B). Furthermore, an *in vivo* metastasis assay demonstrated that overexpression of HRC increased the incidence of lung metastasis and the number of metastatic nodules, but reduced the overall survival. In contrast, the overexpression of CHOP reversed the incidence of lung metastasis and the number of metastatic nodules, whereas prolonged the overall survival of the Huh7-HRC group (Figure 6C-G). These data suggested that ER stress was critical for HRC-enhanced HCC invasion and metastasis *in vivo* and *in vitro*.

## Discussion

Studies have revealed the important role of Ca^2+^-homeostasis in the metastasis of multiple tumors. As the Ca^2+^ regulatory protein, the histidine-rich Ca^2+^-binding protein (HRC), there are indeed few studies on its relationship with anoikis in HCC. In our previous studies, we found that HRC could promote HCC metastasis and growth 8, 9. Hence, the role of HRC in anoikis is of great interest to us. Increasing evidence indicates that anoikis resistance is involved in tumor growth, metastasis and treatment resistance. Resistance to anoikis is an important process of tumor invasion and metastasis 27. It has been proven that enhancing the ability of anoikis resistance can promote the metastasis and progression of HCC 28, 29. Therefore, a better understanding of anoikis resistance in hepatocellular carcinoma cells may contribute to the promising development of therapeutic strategies for malignant tumors. In this study, we demonstrated the association of HRC with anoikis resistance and metastasis in HCC. HRC contributed to the survival of cells under non-anchored conditions and increased the migration capability of cells* in vitro and in vivo*. The remarkable metastatic function of HRC showed that it may be a promising intervention strategy of HCC.

Increasing research in cancer biology has clarified that changes in the level of calcium binding protein are a significant mechanism for regulating the metastasis of HCC cells. S100A8 has been reported to promote cell migration and invasion and EMT in colorectal cancer 30. Silencing the expression of S100A9 partially blocked the growth and metastasis of HepG2 cells induced by HBX *in vitro* and *in vivo* in HCC 31. We demonstrated that exogenous expression of HRC could enhance the invasion and migration of Huh7 cells cultured in suspension, while down-regulation the level of HRC in LM3 cells had the opposite result. Resistance to anoikis is one of the mechanisms that act on the enhancement of cancer cell metastasis 28, 29. To understand whether this mechanism is related to the effect of HRC on metastasis, we studied the regulation of HRC on cell anoikis. In our study, we demonstrated that HRC decreased the rate of anoikis and down-regulation of the expression of HRC could increase the proportion of anoikis. In addition, HRC reduced the level of pro-apoptotic cleaved-caspase3 but increased in anti-apoptotic protein Bcl-2. These data supported the role of HRC in promoting anoikis resistance and served as a potential mechanism for the enhancement of the metastatic capacity in HCC.

Studies have shown that HRC interacts with RyR and SERCA to mediate the of cardiac SR Ca^2+^-uptake and release 32. As our previous studies have shown that HRC inhibits SERCA2, promoting the HCC progression 8, so we tested the expression of proteins RyR1, IP3R3 and SERCA2, which controlled the Ca^2+^-uptake and release. The results showed that HRC significantly decreased the level of SERCA2 when anchoring-independent, while the other two had no obvious changes. We further confirmed when RNA interference (RNAi) was used to inhibit SERCA2 in HCCLM3 cells, the rate of anoikis had increased. In addition, the usage of RNAi for SERCA2 reversed the high anoikis rate caused by low HRC expression. We also found that interfering with SERCA2 can affect the expression of CHOP.

Endoplasmic reticulum (ER) stress is responsible for the oncogenic transformation and tumor growth and metastasis 12, 14. Coordinating the endoplasmic reticulum stress response is a highly dynamic process that can lead to pro-survival and pro-apoptotic outputs. The self-adaptation process when subjected to endoplasmic reticulum stress is named the unfolded protein response (UPR), which is to restore protein homeostasis 33. The UPR has three transmembrane sensor protein pathways, including inositol-requiring enzyme 1 (IRE1), protein kinase RNA-like ER kinase (PERK), and activating transcription factor 6 (ATF6) pathways 34. When the self-protection mechanism of UPR fails, the activation of ATF-CHOP can induce the apoptosis pathway 35. In our study, these data supported that HCC inhibited the induction of ER stress and enhanced anoikis resistance by PERK/ATF4/CHOP signaling pathway. HRC enhanced tumor metastasis progression by inhibiting ER stress *in vitro and in vivo*. Moreover, usage of thapsigargin (TG) to induce ER stress promoted anoikis, while blocking ER stress with 4-phenylbutyrate acid (4-PBA) attenuated anoikis. Sustainability, we will continue to investigate more genes related to ER stress and verify the specific molecular mechanisms between them.

In conclusion, we demonstrated that HRC plays a critical role in protecting HCC metastasis, which dependents on anoikis resistance. We further showed this consequence was mainly mediated by the PERK/ATF4/CHOP signaling pathway dependent on the ER stress (Figure 6H). These findings and considerations highlight the molecular mechanisms underlying HCC and provide a new therapeutic approach for HCC metastasis.

## Supplementary Material

Supplementary tables.

## Figures and Tables

**Table 1:** Correlation Between HRC Expression and Clinicopathological Characteristics in HCC patients.

Clinicopathological variables		Tumor HRC expression		*P* Value
		Negative (n=29)	Positive (n=57)	
Age (year)	<55	14	35	0.260
	≥55	15	22	
Sex	female	5	14	0.585
	male	24	43	
Serum AFP	≤20ng/ml	4	11	0.765
	>20ng/ml	25	46	
Cirrrhosis	absent	6	8	0.539
	present	23	49	
Child-pugh score	Class A	23	49	0.539
	Class B	6	8	
Tumor number	single	24	41	0.303
	multiple	5	16	
Maximal tumor size	≤5cm	10	43	<0.001*
	>5cm	19	14	
Tumor encapsulation	absent	18	19	0.020
	present	11	38	
Microvascular invasion	absent	11	28	0.366
	present	18	29	
TNM stage	I-II	15	45	0.013
	III	14	12	

## Abbreviations

HRC: histidine-rich calcium binding protein
ER stress: endoplasmic reticulum stress
HCC: hepatocellular carcinoma
ECM: extracellular matrix
UPR: unfolded protein response
TNM: tumor-node-metastasis
CHOP: C/EBP homologous protein transcription factor
IHC: immunohistochemical
TCGA: The Cancer Genome Atlas
TG: thapsigargin
4-PBA: 4-phenylbutyrate acid
ATF6: activating transcription factor 6
PERK: PKR-like ER kinase
H&E: Hematoxylin and eosin staining
IRE1: inositol-requiring enzyme 1
RYR: ryanodine receptor
IP3R: inositol-1,4,5-trisphosphate receptor
SERCA: sarco/endoplasmic reticulum calcium ATPase
RNAi: RNA interference
