# Supplementary material for: HRC promotes anoikis resistance and metastasis by suppressing endoplasmic reticulum stress in hepatocellular carcinoma
Source: Int J Med Sci. 2021 Jun 26;18(14):3112–24. doi: 10.7150/ijms.60610 (PMC8364458; doi:10.7150/ijms.60610)
Supplement: Supplementary file 1 — Supplementary tables. [file ijmsv18p3112s1.pdf]

Supplementary Table S1. The primary antibodies used in this study.

Antibodies	Catalog#	Provider
HRC	HPA004833	Atlas Antibodies
Caspase3	9662	Cell signaling technology
Bcl-2	15071	Cell signaling technology
Bip	3177	Cell signaling technology
CHOP	5554	Cell signaling technology
PERK	5683	Cell signaling technology
ATF4	11815	Cell signaling technology
IRE1a	3294	Cell signaling technology
ATF6	65880	Cell signaling technology
SERCA2	9580	Cell signaling technology
IP3R3	610313	BD Transduction Laboratories

Supplementary Table S2. Primers For RT-qPCR

HRC	Forward (5′-3′)<br>Reverse (5′-3′)	GGAACAACAGCACTGGAG<br>GTGCTCAGCTGAGTCTTC
BiP (Grp 78)	Forward (5′-3′)<br>Reverse (5′-3′)	CATCACGCCGTCCTATGTCG<br>CGTCAAAGACCGTGTTCTCG
ATF4	Forward (5′-3′)<br>Reverse (5′-3′)	CCCTTCACCTTCTTACAACCTC<br>TGCCCAGCTCTAAACTAAAGGA
CHOP	Forward (5′-3′)<br>Reverse (5′-3′)	GGAAACAGAGTGGTCATTCCC<br>CTGCTTGAGCCGTTCATTCTC
PERK	Forward (5′-3′)<br>Reverse (5′-3′)	ACGATGAGACAGAGTTGCGAC<br>ATCCAAGGCAGCAATTCTCCC
Bcl-2	Forward (5′-3′)<br>Reverse (5′-3′)	GGTGGGGTCATGTGTGTGG<br>CGGTTCAGGTACTCAGTCATCC
ATF6	Forward (5′-3′)<br>Reverse (5′-3′)	CGCCTTTTAGTCCGGTTCTT<br>CCAGTTGGTAACAATGCCATGT
IRE1a	Forward (5′-3′)<br>Reverse (5′-3′)	GCGCTACAGGCGTTACAAATA<br>TCGTCGAATCCTTCTGGAACT
RYR1	Forward (5′-3′)<br>Reverse (5′-3′)	TCACATGTACGTGGGTGTCC<br>CAACAGGATGACGATGACGA
IP3R3	Forward (5′-3′)<br>Reverse (5′-3′)	CTGCCCAAGAGGAGGAGGAAG<br>GAACAGCGCGGCAATGGAGAAG
SERCA2	Forward (5′-3′)	ATGAGATCACAGCTATGACTGGTG<br>GACTTGCACATCTCTATGGTGACTAG

	Reverse (5 ́- 3 ́)	
GAPDH	Forward (5 ́- 3 ́)<br>Reverse (5 ́- 3 ́)	TCATTGACCTCAACTACATGGTTT<br>GAAGATGGTGATGGGATTTC
